# The effect of habit on preventive behaviors: a two-wave longitudinal study to predict COVID-19 preventive behaviors

**DOI:** 10.1080/21642850.2022.2075876

**Published:** 2022-05-18

**Authors:** Shoji Ohtomo, Reo Kimura

**Affiliations:** a College of Interhuman Symbiotic Studies, Kanto Gakuin University, Yokohama, Japan; b School of Human and Environment, University of Hyogo, Himeji, Japan

**Keywords:** COVID-19 preventive behaviors, behavioral intention, behavioral willingness, dual motivation, habit

## Abstract

Objective: The COVID-19 pandemic is a continuing global threat. This study examined the effect of habit on the motivational aspects of COVID-19 preventive behaviors using a dual-motivation model, which hypothesizes that intentional and reactive motivations determine behavior. This study assumes that habit influences behaviors through the antecedents of the model and the interaction effects of intentional motivation × habit and reactive motivation × habit.

Design: This study conducted a longitudinal survey of 300 Japanese participants to predict preventive behaviors two weeks after the first survey. Moreover, it measured past and future COVID-19 self-reported preventive behaviors, attitudes, behavioral intentions, behavioral willingness, subjective and descriptive norms, self-efficacy, behavioral controls, and habits.

Results: The results showed the interaction effects of behavioral intention × habit and behavioral willingness × habit on preventive behaviors in addition to the effect of past behavior. The stronger the effect of habit, the stronger is the effect of behavioral intention and the weaker the effect of behavioral willingness.

Conclusion: The habituation of preventive behaviors strengthens the behavioral intention–behavior consistency. This study suggested that habit is an important factor for overcoming psychological barriers and for establishing preventive behaviors in daily life.

## Introduction

Since the COVID-19 outbreak caused by a new coronavirus called SARS-CoV-2 was reported in Wuhan, China, in December 2019, over 218 million people have been infected and over 4 million people have died as of September 2021 (WHO, 2021c). While several countries with a high rate of vaccination began to ease restrictions (e.g. social distancing and wearing masks), the number of people infected with the virus increased with the spread of the more infectious Delta variant across the world. Consequently, lockdowns or a state of emergency have been re-implemented in Asian countries and Australia (BBC, 2021; Japantimes, 2021), and Los Angeles County reinstated restrictions and recommendations (e.g. wearing a face mask while in indoor public spaces;CNN, 2021). The COVID-19 pandemic is considered as one of the Chemical, Biological, Radiological, Nuclear, and Explosives (CBRNE) group of disasters. As the Centers for Disease Control and Prevention (CDC, 2021) recommends continuous preventive behaviors even after full vaccination, the establishment of preventive behaviors in daily life is an important public health issue to be addressed in the wake of new biological disasters such as COVID-19.

With the prolongation of the COVID-19 pandemic, studies have been conducted on the psychological processes of preventive behaviors. For example, the relationships between risk perception and preventive behaviors (Savadori & Lauriola, 2021) have been examined, and the application of a motivational model of health-related behavior to preventive behaviors (Hamilton, Smith, Keech, Moyers, & Hagger, 2020; Lin et al., 2020; Scholz & Freund, 2021) has been conducted. However, in the domain of health psychology, the intention–behavior gap (Sheeran, 2002; Webb & Sheeran, 2006) is a deterrent to the prediction of health behaviors. Moreover, shortly after the COVID-19 pandemic began, there were opposition movements against restrictions for preventing infections in some countries (e.g. BBC, 2020). As wearing masks and social distancing limits people’s freedom, they may feel psychological resistance to taking preventive action. Hagger, Smith, Keech, Moyers, and Hamilton (2020) examined the intention–behavior gap in social distancing behaviors within the framework of the *dual-phase* model that includes the motivational and volitional phases. The results showed that past behaviors and habits were more influential in guiding social distancing behaviors than the stepwise process from the motivational to the volitional phase. Preventive behaviors may be becoming customary in daily life because the COVID-19 pandemic has continued for a long time. As it is recommended to maintain preventive behaviors even after being fully vaccinated (WHO, 2021b), examining the psychological process of the habituation of preventive behaviors that encourage people to take action without psychological difficulty is important in the context of the long-term nature of the pandemic. Thus, this study focuses on the effects of habituation of preventive behaviors of COVID-19.

The theory of planned behavior (TPB; Ajzen, 1991) is a prominent framework that has been the most frequently cited model to understand the psychological determinants of health-related behavior (Ajzen, 2011; Nosek et al., 2010). According to TPB, attitude (evaluation of the positive and negative consequences of the behavior), subjective norms (perceived expectations of important others approving or disapproving of the behavior), and perceived behavior control (the perceived capacity to perform the behavior) determine behavioral intention, which is a conscious deliberation of a behavioral decision; moreover, behavioral intention guides behavior directly. However, previous studies (Sheeran & Abraham, 2003; Webb & Sheeran, 2006) suggested that the framework of TPB has limited ability to predict behavior as it is premised on deliberative or intentional decisions. Sheeran, Gollwitzer, and Bargh (2013) argued that health-related behaviors are determined not only by intentional motivation based on deliberation but also by unintentional motivation based on automatic reactions.

To improve the predictive power of TPB, the framework of the dual-motivation model (Ohtomo & Hirose, 2007; Ohtomo, Hirose, & Midden, 2010) was proposed. The dual-motivation model assumes that two types of motivation are involved in social behavior, namely, behavioral intention—conscious deliberation leading to intended behavior (similar to TPB) and behavioral willingness (Gibbons, Gerrard, Blanton, & Russell, 1998)—a reaction to a situation leading to unplanned or non-reflective behavior. Behavioral willingness is considered as the unintentional motivation that is elicited by circumstances conducive to impulsive or spontaneous behavior, regardless of the individual’s conscious intention (Gerrard, Gibbons, Houlihan, Stock, & Pomery, 2008; Gibbons, Gerrard, Reimer, & Pomery, 2006). The perspective of the dual-motivation model is effective wherein the behaviors are determined not only by planned behavioral intention but also by reactive behavioral willingness in predicting health-related behavior such as risky sexual activity (Gibbons et al., 1998; Thornton, Gibbons, & Gerrard, 2002); the use of substances such as alcohol, tobacco, and drugs (Gerrard, Gibbons, Vande Lune, Pexa, & Gano, 2002; Gibbons et al., 2004; Zimmermann & Sieverding, 2010); and unhealthy eating (Dohnke, Steinhilber, & Fuchs, 2015; Ohtomo et al., 2010). Thus, from the perspective of dual motivation, behavioral willingness is an important factor involved in the unintentional motivational process (Todd, Kothe, Mullan, & Monds, 2016; Webb & Sheeran, 2006).

Furthermore, the framework of the dual-motivation model has been applied to examine the habituation of behavior on the motivational process. Within health psychology, the term habitual is considered to denote an automatic process whereby behavior is triggered by contextual cues, through the repetition of behavior in a specific context (Gardner, 2015; Orbell & Verplanken, 2010). There is also a distinction between habitually initiated behavior (instigated by habit) and habitually performed behavior (executed by activation of habits). Habituation of behavior is a complex phenomenon that includes habitual initiation and performance (Gardner & Rebar, 2019). A previous study indicated that habituation of behavior weakens the behavioral intention–behavior relationships because behavior is induced automatically without a conscious decision (Danner, Aarts, & de Vries, 2007). The effect of behavioral intention itself is not eliminated by habit. Automatic response overrides behavioral intention because habituation involves a shift from self-control to an external control that is governed by triggers in a behavioral context through the repetition of behavior in the same context (Orbell & Verplanken, 2010). In the study of the dual-motivation model (Ohtomo, 2013), habit influenced self-control and moderated the effects of the motivational process. In particular, a previous study on unhealthy snacking showed that unhealthy habits strengthen the unintentional process and lead to snacking (Ohtomo, 2017). In this context, this study investigates the motivational processes of habituation of COVID-19 preventive behaviors and salient determinants within the framework of the dual-motivation model.

## Purpose of the study

This study adopted the dual-motivation model to examine the effect of new health-related behavioral habits, that is, habituation of COVID-19 preventive behaviors, on the engagement in the behaviors. The next section outlines the hypotheses of the study.

Regarding the establishment of preventive behaviors, in Hong Kong, which had experienced the threat of SARS (severe acute respiratory syndrome) epidemic, people showed a high level of engagement in preventive behaviors during the influenza A/H1N1 pandemic (Lau, Griffiths, Choi, & Lin, 2010). In Australia, the degree of engagement in hygiene behaviors continued to be high for four months after the national restriction in 2020 (Ayre et al., 2021). The study on habit showed that past behavior was a strong predictor of future behavior and behavioral intention was a weak predictor in stable contexts (Ouellette & Wood, 1998). Maltagliati et al. (2021) reported that adopting a new habit of physical activity was a strong predictor of subsequent behaviors, as the situation stabilized during the lockdown period.

A study on the dual-motivation model (Ohtomo, 2013, 2017) indicated that stronger unhealthy habits imply weaker effects of behavioral intention and stronger effect of behavioral willingness behaviors. Moreover, the interaction effects of habit × motivational factors, such as behavioral intention, have been discussed previously (Gardner, Lally, & Rebar, 2020). The interaction effects of habit and motivational factors differ depending on the correspondence of directions between habit and motivations. When both habit and motivation favor behavior performance (e.g. the habit of preventive behaviors and the motivation to perform preventive behaviors), habit supports the enactment of favorable behavioral intentions. When the direction of habits conflicts with motivation (e.g. the habit of preventive behaviors and the motivation for inaction behaviors), habits weaken the effect of motivation on behaviors. In the dual-motivation model, behavioral willingness is assumed to be a reactive motivation that leads to unplanned or impulsive, risky behaviors (Gibbons et al., 1998; Ohtomo et al., 2010). We hypothesized that habit can mitigate the influence of behavioral willingness when people have a strong habit of preventive behaviors because the directions of habit conflict with behavioral willingness (i.e. tendency for inaction toward preventive behaviors). Conversely, we hypothesized that the habit of preventive behaviors can facilitate the enactment of behavioral intention because habit is consistent with behavioral intention (i.e. favoring preventive behaviors). In addition, because habit itself acts as an antecedent of motivational factors (Ohtomo, 2013), it may have a direct effect on both behavioral intention and willingness.

To investigate the habituation of behavior, it is necessary to distinguish between the effect of habit and the effect of past behavior, such as frequency of actions. Verplanken and Orbell (2003) indicated that habit as a psychological construct, is different from behavioral frequency because the behavioral frequency depends on the type of behavior. Habit includes two aspects, namely, the automaticity of initiation of behavior and performance of the behavior, and behavioral frequency is involved in the latter aspect (Gardner, Rebar, & Lally, 2019). Adopting preventive behaviors to address COVID-19 is a new lifestyle habit and the behaviors are performed frequently in daily life. To understand the process of habit, it is necessary to examine whether the automaticity of performance through the repetition of actions or the psychological internalization of instigation of behavior is strong. This study examines both the effects of past behaviors (i.e. behavioral frequency) and psychological habits to reveal the process of habituation of preventive behaviors to address COVID-19. Lin et al. (2020) in their study of preventive behaviors in terms of COVID-19, found a weak relationship between behavioral intention and behaviors. Hamilton et al. (2020) indicated that past behaviors determined social distancing behaviors over and above the other variables. It has been discussed that past behavior predicted future behaviors strongly because of residual effects (Ajzen, 2011; Bamberg, Ajzen, & Schmidt, 2003). Past behaviors reflect the effects of measured and unmeasured determinants in the theoretical model, and if these determinants remain stable over time, past behaviors can contribute to being a strong predictor of future behaviors. Although the increase in the rate of infections varies from time to time, the situation continued to require preventive behaviors to reduce infections. Accordingly, this study hypothesized that past preventive behaviors exert a strong effect on future behaviors. Despite the significant correlation between habit and prior actions, previous studies have shown that habit and past behaviors independently influence behavior (Bamberg et al., 2003; Verplanken, 2006). The interaction effect of habit × past behaviors is unlikely to be found in the study considering that both variables predict behaviors independently.

Regarding the other determinants of the dual-motivation model, attitude is assumed to affect both motivations, namely, behavioral intention and behavioral willingness (Ohtomo & Hirose, 2014). The dual-motivation model examines the influences of social norms that involved both descriptive norms (Cialdini, Reno, & Kallgren, 1990; Perceptions of how most people behaved) and subjective norms (similar to TPB). Ohtomo and Ohnuma (2014) indicated that subjective norms affected behavioral intention whereas descriptive norms had an effect on both behavioral intentions and willingness. A previous study on wearing masks (Nakayachi, Ozaki, Shibata, & Yokoi, 2020) showed that the conformity norm directed by the behavior of most people was a prominent driving force for wearing masks. Such an effect of the descriptive norm has been shown to facilitate behavior when people favor taking action (Ozaki & Nakayachi, 2020). Compliance with social distancing is related to cognitive resources in relation to the cost–benefit decision that underlies an action (Xie, Campbell, & Zhang, 2020). As preventive behaviors themselves are socially desirable for most people, the influence of descriptive norms may strongly direct behavioral motivations. Moreover, previous studies (Ajzen, 2002; Armitage & Conner, 1999) suggested that perceived behavioral control includes two separable aspects, namely, self-efficacy—a perceived ability to perform a behavior—and behavior control—the perceived controllability of environmental constraints on behavior. In the dual-motivation model, self-efficacy and behavior control are related to the behavioral intention and willingness and make a difference in the influence of the motivational process (Ohtomo, 2013). A previous study indicated that the feasibility of an action is related to the decision making process for preventive behaviors for COVID-19 (Thoma, Weiss-Cohen, Filkuková, & Ayton, 2021). This study assumed that self-efficacy and behavior control are determinants of motivational factors. According to Slovic (1999), people’s reactions to risk events, such as COVID-19, are influenced by demographic factors (e.g. gender and age). Pakpour et al. (2021) reported that elder people vulnerable to COVID-19 tended to adhere to preventive behaviors. This study controls demographic factors including gender, age, and the presence or absence of family members who are vulnerable to CBRNE disasters such as COVID-19 (e.g. children, older family, family with underlying medical conditions) to examine the effects of habit on preventive behaviors within the framework of the dual-motivation model.

The study conducted a longitudinal survey in Japan. Lockdowns or states of emergency were declared in East Asia due to the COVID-19 pandemic. In contrast to western countries, certain regions, such as Hong Kong, Taiwan, and Japan, also experienced the threats of SARS and influenza A/H1N1. Thus, people were relatively less reluctant to take preventive behaviors, such as hand washing and mask wearing. These areas are appropriate for examining the habituation of preventive behaviors related to COVID-19. Previous studies reported that the fear of COVID-19 and social influence were related to preventive behaviors (Chang, Hou, Pakpour, Lin, & Griffiths, 2022; Chung et al., 2022; Nakayachi et al., 2020). In Japan, the state of emergency issued in April 2020 was lifted at the end of May. At the time of the survey, the number of positive cases has decreased in October. However, the Japanese government encouraged the public continue taking preventive measures. Thus, the period was appropriate for examining the habituation of continuous preventive behaviors against COVID-19.

## Method

### Procedure

This study implemented a two-wave longitudinal online survey to measure preventive behaviors and determinants. The web survey was implemented by a web survey company, Cross-Marketing Inc. During the first wave, the study measured preventive behaviors for COVID-19 in the previous week, determinants of preventive behaviors, and demographics. Two weeks after the first wave, the study measured preventive behaviors and showed the debriefing of the research. The survey was conducted from the beginning (16th) to the end of October (31st) 2020. The survey was completed when the final number of respondents reached 300. This study was approved by the institutional research ethics committee (no. 2020023).

### Participants

Among the pooled Japanese samples of Cross-Marketing Inc., 300 respondents were recruited based on the 10 segments of gender (male vs. female) × age (20s, 30s, 40s, 50s, and 60 and over). That is, 30 respondents were included in each segment. The study was approved for the non-clinical survey. At the time of recruitment of the respondents, people with underlying medical conditions (e.g. cardiovascular disease, respiratory disease, diabetes) were prevented from participating in the survey by screening items because they are vulnerable to COVID-19 and their preventive behaviors are assumed to be different from those of others.

### Measurements

With two exceptions (preventive behaviors and attitude), respondents were asked to rate items on 5-point scales ranging from 1 (completely disagree) to 5 (completely agree) as follows.

*Preventive behaviors for COVID-19.* Items of preventive behaviors are based on recommended actions by the WHO (2020) and the Ministry of Health, Labor and Welfare (2020). We asked participants ‘how often have you taken the following actions in the past week?’: ‘Washed your hands with soap or sanitized your hands frequently,’ ‘Carefully washed your hands with soap and water for approximately 30 s,’ ‘Washed your hands and face as soon as you get back home,’ ‘Avoided standing right in front of each other during conversation as much as possible,’ ‘Worn a mask when you went out or talked inside if you were close to other people,’ ‘Kept a distance of two meters as much as possible, or at least one meter, between two persons,’ ‘Avoided places where many people are crowded together,’ ‘Ventilated the interior frequently when you were indoors,’ ‘Refrained from traveling to and from places with a high infection rate,’ ‘Checked your health condition every morning.’ Items were rated on a 5-point Likert scale, ranging from 1 (not at all) to 5 (every time). These 10 items for the first and second wave were averaged: the higher the score, the greater the engagement was in preventive behaviors (first wave: *α* = .90, second wave: *α* = .89).

#### Behavioral intention

We asked participants to respond to the statements: ‘I intend to perform preventive behaviors’ and ‘I plan to perform preventive behaviors.’ The two items were averaged to give a behavioral intention mean score (*α* = .93, *r* = .86).

#### Behavioral willingness

The procedure described by Ohtomo (2013) was adapted to evaluate preventive behaviors. We asked participants to respond to the statements: ‘If the current situation continues, you are unlikely to engage in preventive behaviors’ and ‘If the current situation continues, you will forget to perform preventive behaviors.’ The two items were averaged to give a behavioral willingness mean score (*α* = .88, *r* = .79).

#### Attitude

Similar to previous studies (e.g. Ajzen, 1991; Hamilton et al., 2020; Lin et al., 2020), we measured the attitude to preventive behaviors toward COVID-19 using a semantic differential scale. Participants were presented with the statement, ‘For me, performing preventive behaviors toward COVID-19 is … . .’ Four pairs of adjectives were rated, each on a 5-point bipolar scale, that is, good/bad, favorable/unfavorable, useful/useless, and desirable/undesirable. The mean of the four scales produced a composite scale, with a higher rating reflecting a more positive attitude toward preventive behaviors (*α* = .95).

#### Subjective norm

We asked participants to respond to the statements ‘People who are important to me want me to be concerned about COVID-19 infection’ and ‘People who are important to me want me to perform preventive behaviors in response to COVID-19.’ The two items were averaged to give a subjective norm mean score (*α* = .93, *r* = .87).

#### Descriptive norm

We asked participants to respond to the following statements: ‘Most people around me are concerned about COVID-19 infection’ and ‘Most people around me perform preventive behaviors in response to COVID-19.’ The two items were averaged to give a descriptive norm mean score (*α* = .83, *r* = .83).

#### Self-efficacy

We asked participants to respond to the following statements: ‘It is easy for me to perform preventive behaviors’ and ‘If it were entirely up to me, I would be able to perform preventive behaviors anytime.’ The two items were averaged to give a self-efficacy mean score (*α* = .84, *r* = .73).

#### Behavior control

We asked participants to respond to the following statements: ‘For me, there are situations where it is difficult to perform preventive behaviors’ and ‘Performing preventive behaviors is not entirely up to me,’ The mean of the two scales produced a composite scale, with a higher rating reflecting lower controllability of over-performing preventive behaviors (*α* = .68, *r* = .51).

#### Habit

We measured habit using the 12-item Self Report Habit Index (Verplanken & Orbell, 2003), which was applied to assess preventive behaviors. Three sample items are ‘Performing preventive behaviors in response to COVID-19 is something I do frequently,’ ‘Performing preventive behaviors in response to COVID-19 is something I do automatically,’ and ‘Performing preventive behaviors in response to COVID-19 is something I do without having to consciously remember.’ The 12 items were averaged to obtain a habit mean score; high scores indicated a strong habit (*α* = .90).

#### Demographics*^1^*

In addition to demographic information of the survey sample (i.e. gender and age), we asked about the family members who were vulnerable to the CBRNE disasters, including COVID-19. Multiple-answer items and their results pertaining to the whole sample were as follows: babies and infants, primary school children, junior high school students, high school students, people over 65 years, people who need long-term care or assistance, expectant and nursing mothers, and people with underlying medical conditions (e.g. cardiovascular disease, respiratory disease, and diabetes).

### Statistical analysis

To examine the determinants of motivation (i.e. behavioral intention and willingness) and preventive behaviors, a Bayesian Generalized Linear Models with Gaussian distribution was conducted. The model for predicting behavioral intention or behavioral willingness, previous preventive behaviors, attitude, subjective norm, injunctive norm, self-efficacy, behavioral control, habit, age, gender (dummy variable), and presence of a vulnerable family member (dummy variables) were taken as the dependent variables. The model for predicting further preventive behaviors, behavioral intention, behavioral willingness, interaction terms of previous preventive behavior × habit, behavioral intention × habit, and behavioral willingness × habit were added as independent variables. The quantitative variables of interaction terms were mean-centered. The analysis was performed in R (R Core Team, 2016) and the brms package (Bürkner, 2017). In the analyses of the model, all iterations were set to 10,000 and burn-in samples were set to 5,000, with the number of chains set to four. The model confirmed that the value of Rhat for all parameters equaled 1.0, indicating convergence across the four chains. To examine the strength of the associations between variables, maximum *a posteriori* (MAP) and expected *a posteriori* (EAP) of coefficients were reported. According to Kruschke (2014), the interval estimation of coefficients is significant as long as 0 is not included between 95% credible intervals.

## Results

The final sample of 300 was composed of an equal number of male and female respondents with an average age of 45.57 (*SD* = 14.74) years. Family members who were vulnerable to CBRNE disasters were infants and young children (7%), school-aged children (primary = 9%; junior high school = 7%; high school = 6%), people aged over 65 years and who need long-term care or assistance (3%), expectant and nursing mothers (1%), and people with underlying medical conditions (4%). For analysis, the items of the responses were transformed into binary dummy variables with yes (1) and no (0) representing the presence (48%) or absence (52%) of vulnerable family members, respectively.

Table 1 reports the mean, standard deviation, and correlations between the past and future preventive behaviors for COVID-19 and its determinants. First, the result of the model for predicting behavioral intention to take action showed that past preventive behaviors, attitude, self-efficacy, descriptive norm, and habit had an effect on behavioral intention (Table 2).

**Table 1.** Descriptive statistics and correlations among the variables.

		1	2	3	4	5	6	7	8	9	10	11
1	Future preventive behaviors	1.000										
2	Past preventive behaviors	.691**	1.000**									
3	Behavioral intention	.057	.089	1.000								
4	Behavioral willingness	.367**	.425**	.161**	1.000							
5	Age	.379**	.531**	.208**	.613**	1.000						
6	Attitude	-.169**	-.156**	-.092	-.280**	-.316**	1.000					
7	Self-efficacy	.312**	.427**	.185**	.341**	.507**	-.121*	1.000				
8	Behavior control	.222**	.333**	-.009	.189**	.305**	.361**	.565**	1.000			
9	Subjective norm	.226**	.391**	.077	.234**	.348**	.044	.422**	.468**	1.000		
10	Descriptive norm	.296**	.429**	.136*	.368**	.509**	-.134*	.469**	.381**	.483**	1.000	
11	Habit	.410**	.592**	.152**	.445**	.587**	-.096	.598**	.464**	.485**	.500**	1.000
M		3.802	3.729	4.242	2.538	45.573	4.160	3.662	3.178	3.300	3.783	3.436
SD		.811	.878	.836	1.056	14.740	.865	.935	.757	1.001	.882	.666

* *p *< .05, ** *p *< .01

**Table 2.** Results of the Bayesian GLM for motivation variables.

	Behavioral intention						
	MAP	EAP	sd	95%LCI	95%UCI	n_eff	Rhat
Intercept	.403	.414	.225	-.027	.855	21627	1.000
Past preventive behaviors	.139	.143	.048	.047	.238	22276	1.000
Age	.004	.004	.002	-.001	.008	28948	1.000
Gender(vs.female)	.117	.123	.068	-.009	.256	23948	1.000
Vulnerable people(vs.yes)	.012	.000	.066	-.131	.130	24273	1.000
Attitude	.324	.329	.044	.242	.416	22869	1.000
Self-efficacy	.147	.143	.049	.047	.238	20185	1.000
behavior control	-.034	-.038	.055	-.147	.071	21611	1.000
Subjective norm	-.005	-.011	.041	-.092	.070	21980	1.000
Descriptive norm	.139	.146	.048	.052	.241	21997	1.000
Habit	.227	.227	.073	.083	.372	19572	1.000
	Behavioral wiingness						
	MAP	EAP	sd	95%LCI	95%UCI	n_eff	Rhat
Intercept	2.881	2.877	.333	2.226	3.535	23532	1.000
Past preventive behaviors	-.126	-.125	.072	-.265	.016	26250	1.000
Age	.002	.002	.004	-.004	.009	27744	1.000
Gender(vs.female)	-.056	-.044	.103	-.245	.158	26754	1.000
Vulnerable people(vs.yes)	.181	.185	.100	-.012	.379	28803	1.000
Attitude	-.271	-.266	.068	-.399	-.133	26621	1.000
Self-efficacy	-.377	-.373	.075	-.519	-.226	25316	1.000
behavior control	.928	.923	.085	.757	1.090	25312	1.000
Subjective norm	.024	.019	.062	-.104	.142	24506	1.000
Descriptive norm	-.144	-.152	.071	-.292	-.013	26233	1.000
Habit	-.009	.001	.111	-.220	.220	25256	1.000

Note: MAP = Maximum A Posteriori, EAP = Expected A Posteriori, LCI = Lower limits of Credible Interval, and UCI = Upper limits of Credible Interval.

Second, the result of the model for predicting behavioral willingness, that is, the reaction not to take action showed that attitude, self-efficacy, behavioral control, and descriptive norm had effects on behavioral willingness (Table 2).

**Figure 1.**
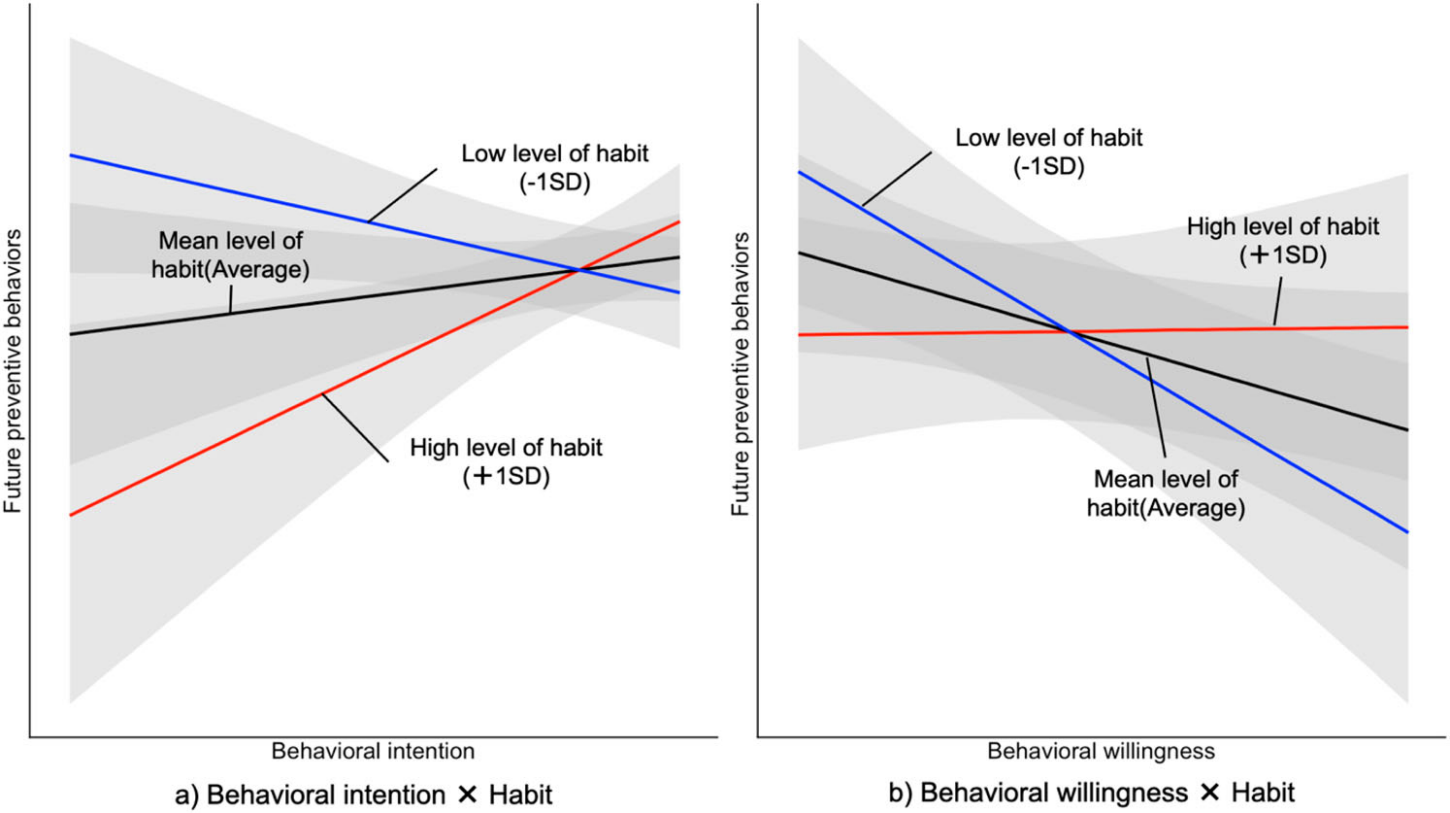
Plots of interaction effects of behavioral intention × habit and behavioral willingness × habit on future preventive behavior. Note: For example, the effect of behavioral intention on behavior with high levels of habit (+1 SD) was b_MAP_ = .263 (b_EAP_ = .248; 95% CI = .065 to .435), with a mean level of habit (average) at b_MAP_ = .063 (b_EAP_ = .065; 95% CI = −.065 to .195), and a low level of habit (−1 SD) at b_MAP_ = −.113 (b_EAP_ = −.117; 95% CI = −.238 to .003). Moreover, the effect of behavioral willingness on behavior with high levels of habit (+1 SD) was b_MAP_ = .004 (b_EAP_ = .003; 95% CI = −.079 to .085) with the mean level of habit (average) at b_MAP_ = −.075 (b_EAP_ = −.073; 95% CI = −.151 to .005) and a low level of habit (−1 SD) at b_MAP_ = −.159 (b_EAP_ = −.148; 95% CI = −.248 to −.049).

Finally, the result of the model for predicting future preventive behaviors showed that past preventive behaviors, gender, interaction of behavioral intention × habit, and interaction of behavioral willingness × habit had an effect on future preventive behaviors (Table 3). Figure 1 plots the posterior distribution of these interaction effects by Bayesian estimation. Concerning the interaction effect of the behavioral intention × habit, when people had strong habits, high intention led to an increase in preventive behaviors (Figure 1a). When people had weak habits, they were less likely to perform preventive behavior even if they had strong intentions. Concerning the interaction effect of behavioral willingness × habit, when people had weak habits, high behavioral willingness led to a decrease in preventive behaviors (Figure 1b). When people had strong habits, they tended to be less susceptible to the influence of behavioral willingness.

**Table 3.** Results of the Bayesian GLM for future preventive behaviors.

	MAP	EAP	sd	95%LCI	95%UCI	n_eff	Rhat
Intercept	3.446	3.445	.322	2.832	4.082	30955	1.000
Past preventive behaviors	.616	.613	.051	.511	.713	26790	1.000
Age	-.0002	.000	.002	-.005	.004	37332	1.000
Gender(vs.female)	.204	.203	.066	.074	.332	35345	1.000
Vulnerable people(vs.yes)	.123	.116	.064	-.013	.241	35250	1.000
Behavioral intention	.063	.065	.066	-.065	.195	24339	1.000
Behavioral willingness	-.075	-.073	.040	-.151	.005	24780	1.000
Attitude	.057	.053	.048	-.042	.145	31255	1.000
Self-efficacy	.016	.019	.051	-.081	.118	27555	1.000
behavior control	.038	.045	.065	-.081	.172	24749	1.000
Subjective norm	-.050	-.055	.040	-.134	.023	34017	1.000
Descriptive norm	-.034	-.031	.046	-.122	.060	34106	1.000
Habit	-.026	-.026	.073	-.170	.120	32250	1.000
Past preventive behaviors × Habit	-.020	-.019	.067	-.151	.110	21341	1.000
Behaviral intention× Habit	.279	.271	.068	.139	.404	20050	1.000
Behaviral willingness × Habit	.112	.114	.036	.044	.184	34113	1.000

Note: MAP = Maximum A Posteriori, EAP = Expected A Posteriori, LCI = Lower limits of Credible Interval, and UCI = Upper Limits of Credible Interval.

## Discussion

Due to the emergence of the highly contagious Delta variant of COVID-19, the number of infected people has increased sharply worldwide, and even countries with high vaccination rates have required people to take preventive action again (e.g. CDC, 2021). In a situation wherein the threat of COVID-19 is prolonged, it is important to root preventive behaviors in people. Studying the process of the establishment of preventive behaviors such as habits is an important issue in the current pandemic situation. This study examines the effects of the habitual process with a dual-motivation model and reveals psychological factors that root preventive behaviors in people.

First, the behavioral intention to take preventive action was influenced by past preventive behaviors and habits. In a study of social distancing behavior (Hamilton et al., 2020), past behaviors had a strong effect on behavioral intentions. A previous study indicated that habit influenced behavioral intention over and above the past behaviors and attitudes (Bamberg et al., 2003). Thus, the habit of practicing desirable behaviors (e.g. using public transport or performing preventive behaviors) can affect the behavioral intention as a decision heuristic to instigate behavior. Attitude and self-efficacy promotes behavioral intention and consistent with previous studies of the motivational model (Ajzen, 1991; Ohtomo, 2013), these variables can affect the behavioral intention of preventive behaviors. In addition, the descriptive norm, through perceptions of how most people behaved, promoted behavioral intention. In a study on the use of masks against COVID-19 (Nakayachi et al., 2020), conformity to the social norm promoted wearing masks. The effect of the descriptive norm on behavioral intention was found in the study on socially desirable behaviors (Ohtomo & Ohnuma, 2014). Previous studies on preventive behaviors indicated that attitude and social norm were important factors orienting people’s behavior (Aschwanden et al., 2021; Savadori & Lauriola, 2021).

Second, behavioral willingness that leads the individual to not take preventive action was not influenced by habit, but by previous preventive behaviors, and self-efficacy and behavioral control affected behavioral willingness. Thus, people who practice repeated preventive behaviors can respond automatically in terms of behavior and decrease the tendency to inaction. Another previous study on unhealthy eating (Ohtomo, 2013) indicated that self-efficacy inhibited behavioral willingness, and behavioral control promoted behavioral willingness. Furthermore, self-efficacy was found to be an important factor in continuous preventive behaviors (Ayre et al., 2021). People who were not habituated to preventive behaviors could have weak intrinsic self-efficacy and their behaviors are likely to depend on extrinsic behavior control. Behavioral willingness that is a reaction to behavioral contexts could be promoted when self-control was depends on behavioral control rather than self-efficacy; moreover, attitude and descriptive norms influenced behavioral willingness; consistent with extant studies on the dual-motivation model (Ohtomo & Hirose, 2014; Ohtomo & Ohnuma, 2014), these factors can orient behavioral willingness. In particular, normative influences were important for predicting preventive behaviors in response to COVID-19 (Hagger et al., 2020; Nakayachi et al., 2020; Savadori & Lauriola, 2021). The descriptive norms could be a function of behavioral contexts that orient motivations.

Third, past preventive behaviors strongly affected future behaviors. Studies on social distancing indicated that past behaviors had a strong effect on future behaviors over and above other variables (Hagger et al., 2020; Hamilton et al., 2020). Because the COVID-19 pandemic has continued for a long time, past behaviors can be an important predictor of behavior as residual effects (Ajzen, 2011; Bamberg et al., 2003) that include the stability of various behavioral decisions. Although the COVID-19 pandemic induced a context change of behavior and weakened the relationships between previous habits and behavior, new habits began to control behaviors due to the prolongation of the pandemic (Maltagliati et al., 2021). In addition, gender effects showed that women took preventive measures more than men did. Generally, women are more concerned with safety than men (Slovic, 1999), and such a tendency might be expressed in preventive behaviors. Previous studies also indicated that demographics led to a difference in the adherence to preventive behaviors (Pakpour et al., 2021; Savadori & Lauriola, 2021). Moreover, the interaction effects of behavioral intention × habit and behavioral willingness × habit indicated that the stronger the habit, the stronger the behavioral intention and the weaker the effect of behavioral willingness on preventive behaviors. In other words, the more habitual the preventive behaviors become, the more consistent the behavioral intention and behavior become in turn. Gardner et al. (2020) posited that habit strengthens the relationship between behavioral intention and behavior when habit and intention both favor behaviors. When behaviors are habitualized, the cognitive efforts of the execution of behaviors are weakened and intended behaviors become easier to execute (Gardner et al., 2019). Furthermore, as the behavioral willingness that leads to unintentional inaction in terms of preventive behaviors is suppressed, other behaviors are less likely to occur when preventive behaviors are habituated. Habit has been considered a significant barrier to behavior change (Verplanken, 2006). Habituation of preventive behaviors can induce shield effects (Danner et al., 2007) against the influence of motivation to unintentional inaction and intended behaviors can be promoted. This study suggests that the habit of preventive behaviors moderates the effects of dual motivation and promotes the behaviors indirectly, rather than directly, when the behavioral contexts under the COVID-19 pandemic conditions are stable.

## Limitations

This study includes several limitations. The necessity of preventive behaviors in response to COVID-19 changes depending on the situation. Even now that vaccination is widespread in some countries, the number of infections is increasing worldwide; hence, the importance of preventive behaviors is increasing. Considering that more than a year has passed since COVID-19 emerged, it is required that preventive behaviors become customary in daily life. Therefore, further research that examines changes in event-based long-term preventive behaviors, such as the waves of pandemics, lockdowns (states of emergency), and vaccination, are needed. Additionally, there are differences in the prevalence of COVID-19 between nations and between regions. People in regions that experienced the threats of SARS and influenza A/H1N1 were relatively familiar with preventive behaviors for infectious diseases (Chang et al., 2022; Chung et al., 2022; Lau et al., 2010). Accordingly, an examination of geopolitical contexts is required to improve the validity of the prediction model. Moreover, the new mutation (i.e. Omicron variant) emerged as a new threat (WHO, 2021a), and the number of infected people increased worldwide. Although countries with high rates of booster vaccination lifted restrictions, the number of infected people continued to increase in certain regions, such as East Asia (e.g. CNN, 2022). In the future, the emergence of new mutations and the pandemic scenarios may change the measure of restrictions undertaken by the government and the habit of preventive behaviors of the people. From the perspective of measurement, this study noted a technical limitation in the compatibility of the measurement between preventive behaviors and determinants, which is similar to Lin et al. (2020). We measured preventive behaviors using 10 specific items and determinants, such as habit, attitude, and behavioral intention as general items. Thus, the correlations between future preventive behaviors and determinants are relatively weak compared with those between past and future preventive behaviors. This study measured preventive behaviors with self-reporting. The self-reported data are at the risk of self-presentation bias and socially desirable responses. Further research should consider the use of a more objective approach, such as an ecological momentary assessment to measure behaviors accurately.

## Conclusion

This study examined the effects of past behaviors and habits within the framework of the dual-motivation model to predict preventive behaviors in response to COVID-19. The findings reveal that stable context enhanced the predictive power of previous preventive behaviors on future behaviors, and the habit of preventive behaviors strengthened the effect of intentional motivations that led to the behaviors and suppressed the effects of the reactive motivations that inhibited the behaviors. Thus, habituation is important to promote intention–behavior consistency, leading to preventive behaviors. Moreover, attitude, descriptive norms, self-efficacy, and behavioral control affected the two motivations. These factors might be important to form the motivations before habituation of preventive behaviors. As COVID-19 is one of the CBRNE disasters, the application of a model of health-related behaviors can be an available measure to fight new infection disasters.

## Data Availability

A questionnaire, data files, and output of analyses are available online: https://osf.io/23nq9/?view_only=9ffb03d244b24cd9b2884575f57a0475

## Note

1 The study measured the preparations for disasters at home as a part of our research project. We did not use it in analysis because it is not directly related to our model of the study.
